# Inhibition of the IRE1/JNK pathway in renal tubular epithelial cells attenuates ferroptosis in acute kidney injury

**DOI:** 10.3389/fphar.2022.927641

**Published:** 2022-08-26

**Authors:** Yan Liang, Zhenjie Liu, Lingyun Qu, Yingzi Wang, Yali Zhou, Lulu Liang, Yanhong Guo, Lin Tang

**Affiliations:** ^1^ Department of Nephrology, The First Affiliated Hospital of Zhengzhou University, Zhengzhou, China; ^2^ Research Institute of Nephrology, Zhengzhou University, Zhengzhou, China; ^3^ Key Laboratory of Precision Diagnosis and Treatment for Chronic Kidney Disease in Henan Province, Zhengzhou, China

**Keywords:** AKI (acute kidney injury), IRE1 (inositol-requiring enzyme 1), JNK (c-Jun N-terminal kinase), ferroptosis, ER stress, tubular epithelial cells

## Abstract

**Backgroud:** Ferroptosis is a form of regulated cell death in ischemia-reperfusion (I/R) injury models. Acute kidney injury (AKI) induced by I/R injury can result in cell death, and subcellular structural changes, including expansion of the endoplasmic reticulum (ER), mitochondrial shrinkage, and other morphological changes. Inositol requiring enzyme 1 (IRE1) a proximal ER stress sensor, activates c-Jun NH2-terminal kinases (JNK) in response to ER stress, which is inextricably linked to ER.

**Method:** To determine the resulting damage and relationship between ferroptosis and the IRE1/JNK pathway in AKI, we modeled AKI in I/R renal injury mice and hypoxia/reoxygenation (H/R) HK-2 cells, as *in vivo* and *in vitro* experiments, respectively.

**Results:** In I/R renal injury mice, we found that abnormal renal function; damage of renal tubular epithelial cells; activation of the IRE1/JNK pathway and ferroptosis. Our *in vitro* study showed a large number of reactive oxygen species and more ferroptotic mitochondria in H/R HK-2 cells. By inhibiting IRE1/JNK in I/R renal injury mice, we observed decreased blood urea nitrogen, creatinine, and tissue injury, compared with the I/R group, we also found the markers of ferroptosis changed, including decreased 4-hydroxynonenal and increased glutathione peroxidase 4, as well as in H/R induced IRE1/JNK knock-down HK-2 cell lines (stable depletion). Furthermore, inhibition of ferroptosis could also attenuate the IRE1/JNK pathway in mice following I/R and HK-2 cells following H/R.

**Conclusion:** We observed cross-talk between the IRE1/JNK pathway and ferroptosis in I/R or H/R induced AKI. Our findings suggest that ferroptosis plays an important role in I/R induced AKI, and that inhibition of the IRE1/JNK pathway can protect against I/R induced renal injury by inhibiting ferroptosis. The inhibition of the IRE1/JNK pathway could therefore be a feasible therapeutic target for treatment of AKI.

## Introduction

Acute kidney injury (AKI) is a common disease worldwide, and has a particularly high incidence among intensive care unit patients (Chen et al., 2017). AKI associated mortality remains high, as greater than one in four patients with AKI died within 1 year of hospitalization (Sohaney et al., 2022). AKI is pathologically characterized by sublethal and lethal damage of renal tubules. In our previous study, we reported that the inositol-requiring enzyme 1 (IRE1)/c-Jun NH2-terminal kinase (JNK) pathway in the endoplasmic reticulum (ER) stress is activated in cases of AKI to mediate renal tubules epithelial cell damage incurred from inflammatory reactions (Liang et al., 2020). Renal tubular cell death is an early symptom of AKI, characterized by abnormalities of the ER, mitochondria, and other organelles through the release of chemokines from acutely injured cells that can induce multiple cell death pathways, such as apoptosis and ferroptosis.

Ferroptosis is an iron–dependent, regulated necrotic subroutine characterized by increased lipid peroxidation resulting from a lack of glutathione, which is critical to the functioning of glutathione peroxidase. This type of cell death is morphologically characterized by mitochondrial shrinkage and reduction of mitochondrial crista in injured cells (Xie et al., 2016). Recent studies have suggested that ferroptosis is involved in ischemia-reperfusion (I/R) injury, whereas necroptosis is more commonly observed in nephrotoxic AKI (Linkermann et al., 2014). Ferroptosis is induced by the activation of mitochondrial, voltage-dependent anion channels and mitogen-activated protein kinases that contribute to the upregulation of ER stress and inhibition of cystine/glutamate antiporters (Hong et al., 2017). Some studies have referred to this phenomenon as “cross talk” between ER stress and ferroptosis (Chen et al., 2017; Hong et al., 2017; Lee et al., 2019; Park et al., 2019; Zhang et al., 2019; Li et al., 2020; Zhao et al., 2021). It is thought that the JNK signaling pathway may be involved in the induction of ferroptosis, through the activity of SP 600125, a JNK inhibitor that also inhibits erastin, a notable inducer of ferroptosis (Heslop et al., 2020). Inhibition of JNK under hypoxia could prevent mitochondrial ferritin induction and protect from ferroptosis in macrophages (Fuhrmann et al., 2020). Moreover, some reports showed ferroptosis plays an important role in inflammation (Sun et al., 2020). Probably, there is a relationship between IRE1/JNK pathway in ER stress and ferroptosis in AKI, which is few reported and requires further study.

In this study, we assessed renal function, subcellular morphology, activation of the IRE1/JNK pathway, and the production of reactive oxygen species (ROS) as potential biomarkers of ferroptosis in mice with I/R-induced renal injury and the human renal proximal tubular epithelial cell line (HK-2 cells) with induced hypoxia/reoxygenation (H/R). We further investigated the role of the cross-talk between the IRE1/JNK pathway and ferroptosis in I/R-induced mice, and in H/R induced knockdown HK-2 cells. The results of this study will contribute to understand the roles of the IRE1/JNK pathway and ferroptosis within the pathological mechanism of renal tubule injury in AKI, which could be a target for therapeutic treatment of AKI.

## Materials and methods

### Mouse renal ischemia-reperfusion model

One hundred and forty-four healthy male, eight-weeks-old C57BL/6J mice (20–25 g in mass) were purchased from Beijing Vital River Laboratory Animal Technology Co., Ltd. (Beijing, China) and kept in the animal experimentation center at the Medical Science Research Institute in the Henan Province (Zhengzhou, China). According to the experimental design, there were six mice in each group. The mice were given sufficient food and water and kept at 25°C and 50% relative humidity for 2 weeks. To establish the I/R model, the mice received intraperitoneal injections of pentobarbital sodium (50 mg/kg) as previously described (Park et al., 2003). A dorsal midline laparotomy and bilateral renal artery occlusion were then performed on each mouse on a 37.5°C heating plate. The vascular clamp was applied to reduce blood flow, and 27 min removed, then the kidney had turned black and red. The perfusion was then restored after the kidney gradually returned to a solid red color. The mice were euthanized at 12, 24, 48, or 72 h after perfusion, after which the kidneys were harvested and blood samples were collected. Both kidneys were collected and perfused from the heart with precooled normal saline, then dissected and fixed in 10% neutral buffered formalin or rapidly stored in liquid nitrogen for later use. As the sham group, the mice underwent the same surgical procedure, except for arterial occlusion. The groups were called the Sham (12, 24, 48, 72 h) and the I/R (12, 24, 48, 72 h) group, respectively. All procedures were carried out in accordance with the institutional animal care guidelines and approved by the ethics committee of the First Affiliated Hospital of Zhengzhou University.

### Inhibitor treatment

The Irestatin 9389 IRE1 inhibitor (626221-47-4, Axon Medchem, Netherlands) and the SP 600125 JNK inhibitor (1496, TOCRIS, United States) were prepared per the manufacturers’ instructions; either 50 mg/kg Irestatin 9389 or 15 mg/kg SP 600125 was administered to the mice by intraperitoneal injection at 24 and 2 h pre-operation, which were designated as the I/R + Irestatin 9389 (12, 24, 48, 72 h) and I/R + SP 600125 (12, 24, 48, 72 h) group, respectively. Ferrostatin-1 (Fer-1, 0.8 mg/kg, Sigma-Aldrich Chemical, Darmstadt, Germany), a ferroptosis inhibitor, was dissolved in DMSO and 0.9% NaCl and injected into mice via tail vein at 24 h before I/R. The I/R and control groups were given an intraperitoneal injection of normal saline.

### Renal function test

Before perfusion with normal saline, a blood sample was collected from the heart of each mouse. The sample was then centrifuged at 4°C, 5000 rpm for 10 min. The supernatant was collected and analyzes using an automatic biochemical analyzer (Beckman Coulter, United States).

### Electron microscopic processing of renal tissues

Tissue samples from the kidneys were perfused with normal saline then fixed with glutaraldehyde, washed with phosphate buffer saline (PBS), fixed with osmium acid, and washed again with PBS. After 24 h, the samples were subjected to gradient dehydration, and resin permeation polymerization, after which ultrathin sections (50 nm) were sliced. The sections were stained with uranyl acetate and lead citrate and observed under a transmission electron microscope (JEOL, Tokyo, Japan), where in five fields of view were randomly selected for each sample for analysis.

### Periodic Acid-Schiff staining

Kidney tissue samples were fixed with 4% paraformaldehyde, embedded in paraffin, and processed into 4-µm thickness sections. The sections were then gradually deparaffinized and hydrated and stained with Periodic Acid-Schiff (PAS) staining, which was performed per the manufacturers’ protocol. For each kidney 100 cortical tubules from at least 10 different areas were scored as described by Paller et al. (Paller et al., 1984). The results showed the following results: evident renal tubule expansion and flat cells (1); the appearance of tube-type cells in renal tubules (2); the appearance of some exfoliated necrotic cells in the lumen of the renal tubules but not tubular or cell fragments (1); vacuolar degeneration (1); and karyopyknosis (1).

### Western blotting of renal tissues

Renal tissues were homogenized in RIPA buffer (P0013, Beyotime, Shanghai, China) supplemented with protease inhibitors (539134, EMD Biosciences Inc., San Diego, CA). After centrifugation (12,000 rpm/min) at 4 °C for 15 min, the supernatant was added to the loading buffer. Protein samples were processed for immunoblot analysis as previously reported (Dixon et al.). The following primary antibodies were used: β-actin (AP0060; Bioworld Technology), protein kinase -like endoplasmic reticulum kinase (PERK, ab65142; Abcam), activating transcription factor 6 (ATF6, ab203119; Abcam), IRE1α (CST3294), p-IRE1α (phosphorylation sites: S724; ab124945; Abcam), JNK (CST9252), p-JNK (phosphorylation sites: Thr183/Tyr185; CST4668), CCAAT-enhancer-binding protein-homologous protein (CHOP, ab179823; Abcam), 4-hydroxynonenal (4-HNE, ab46545; Abcam), glutathione peroxidase 4 (GPX4, ab125066; Abcam). The working concentration of β-actin used was 1:5000, and that of the other antibodies was 1:1000. Secondary antibodies were obtained from Dingguo Co. Ltd. (Beijing, China) and were used at the final dilution of 1:5000. The odesay software was used for analysis (Li Cor, Lincoln, NE).

### Hypoxia/reoxygenation model of HK-2 cells

HK-2 cells were purchased from ATCC (the Global Bioresource Center) and cultured at 37 °C and 5% CO_2_ in a humidified incubator with Dulbecco’s Modified Eagle Medium (DMEM)/F12 medium (Gibco, Thermo Fisher Scientific, United States), which contains 10% fetal bovine serum (FBS, Gibco), 100 U/mL penicillin, 100 μg/ml streptomycin, and 10% fetal bovine serum (Geneview), 1% non-essential amino acids (M7145, Sigma, Missouri, United States), and 1% glutamine amide (G3126, Sigma). The methods used to establish the H/R injury model are described in detail in our previous study (Liang et al., 2020). Briefly, HK-2 cells with serum-free DMEM/F12 were equilibrated to 37°C, and subjected to hypoxic conditions of 5% CO_2_, 1% O_2_, and 94% N_2_ for 4 h. The cells were harvested at 6, 12, and 24 h after culturing the cells under normal oxygen content conditions of 5% CO_2_ and 95% air at 37°C. The control group was incubated in a humidified incubator with 5% CO_2_ at 37°C across all processes.

### HK-2 stable transfection cell lines

IRE1α or JNK1 knock-down HK-2 cell lines (stable depletion) were generated by the Obio Technology Corp. Ltd. (Shanghai, China), as described in our previous study (Liang et al., 2020). The two stable cell lines were cultured in DMEM/F12 medium. In some experiments, these cells were subjected to H/R treatment.

### Inhibition of ferroptosis in HK-2 cells

To study the effect of ferroptosis on IRE1/JNK pathway in AKI, Fer-1 was added to HK-2 cells media at a concentration of 10 mmol/L, continuously cultured for 24 h before H/R (Baba et al., 2018).

### Cell electron microscope processing

Following H/R, HK-2 cells were trypsinized and centrifuged at 1000 rpm for 10 min. Thereafter, cells were soaked with 2% glutaraldehyde for a minimum of 1 h at 4 °C. Cells were then thoroughly washed with 0.1 mol/L PBS, without disrupting the cell pellet. Following decanting of the supernatant, 1% osmium acid (in PBS) was added and the mixture left standing for 1.5 h. Following another washing step, cells were dehydrated with increasing concentrations of acetone (30, 50, 70, 90 and 100%) and embedded in 812 resin (Canemco, 034) over night. Cells were then observed under a transmission electron microscope (JEOL). Five fields of view were randomly selected for each sample, and the average of the five samples were used for analysis.

### Western blotting of HK-2 cells

HK-2 cells were lysed and subsequently treated, which was the same as it in the renal tissue of mice. The primary antibodies and second antibodies were used as in the Western blotting of renal tissue.

### RNA extraction and quantitative reverse transcription-polymerase chain reaction

Total RNA was extracted from cells using a commercial RNA Extraction Kit (Qiagen) according to the manufacturer’s protocol and cDNA was synthesized (from RNA templates) using the Revert Aid First Strand cDNA Synthesis Kit (Thermo Fisher Scientific). RNA extraction and quantitative reverse transcription-polymerase chain reaction (qRT-PCR) was performed in triplicate using the Maxima SYBR Green qPCR Master Mix (2X) with a separate ROX vial (Thermo Fisher Scientific) and the SLAN-96P Real-Time PCR System. The mRNA levels were normalized to those of the housekeeping gene actin-beta. The primers used are listed in Table 1.

**TABLE 1:** Primer sequences used in this study (qRT-PCR).

Gene	Primer sequence
**CHOP**	Forward 5′-GGT​ACC​TAT​GTT​TCA​CCT​CCT​GG-3′
	Reverse 5′-CTC​CTC​AGT​CAG​CCA​AGC​CA-3′
**IRE1α**	Forward 5′-GCA​AGA​GGA​CAG​GCT​CAA​TCA-3′
	Reverse 5′-GAT​TCC​ATC​TGA​ACT​TCG​GCA-3′
**JNK1**	Forward 5′-AGC​CAG​TCA​GGC​AAG​GGA​TT-3′
	Reverse 5′-ATT​GAT​GTA​CGG​GTG​TTG​GAG​A-3′
**GPX4**	Forward 5′-CCA​GTG​AGG​CAA​GAC​CGA​AGT-3′
	Reverse 5′-TCC​TGC​TTC​CCG​AAC​TGG​TTA-3′

### ROS detected by flow cytometry

ROS levels were measured using the ROS determination kit (Wan Lei Biotechnology Co. Ltd., Shenyang, China). HK-2 cells were collected, then centrifuged at 300 g for 5 min. The supernatant was carefully aspirated, the cells were washed once with PBS, and then centrifuged again at 300 g for 5 min to collect cells in each group. One milliliter of 2,7-Dichlorodi -hydrofluorescein diacetate (DCFH-DA) diluent was added to the supernatant, mixed well, and incubated in at 37°C for 30 min. For thorough mixing, sample tubes were turned upside down every three to 5 minutes. The cells were washed three times with PBS to fully remove the DCFH-DA that did not permeate the cells. The cells were resuspended in PBS, and flow cytometry was performed (NovoCyte, ACEM, United States).

### Statistical analysis

A two-tailed Student’s t-test was performed to compare means between two groups. A one-way analysis of variance was performed for comparisons between more than two groups. Each experiment was performed to confirm the results. Statistical analysis was performed using GraphPad Prism 5.0 (GraphPad Prism Software, CA, United States). For all analyses, a *p*-value < 0.05 was considered statistically significant. All values are expressed as the mean ± SD.

## Results

### I/R renal injury leads to abnormal renal function, ER stress in renal tubules of mice

**FIGURE 1:**
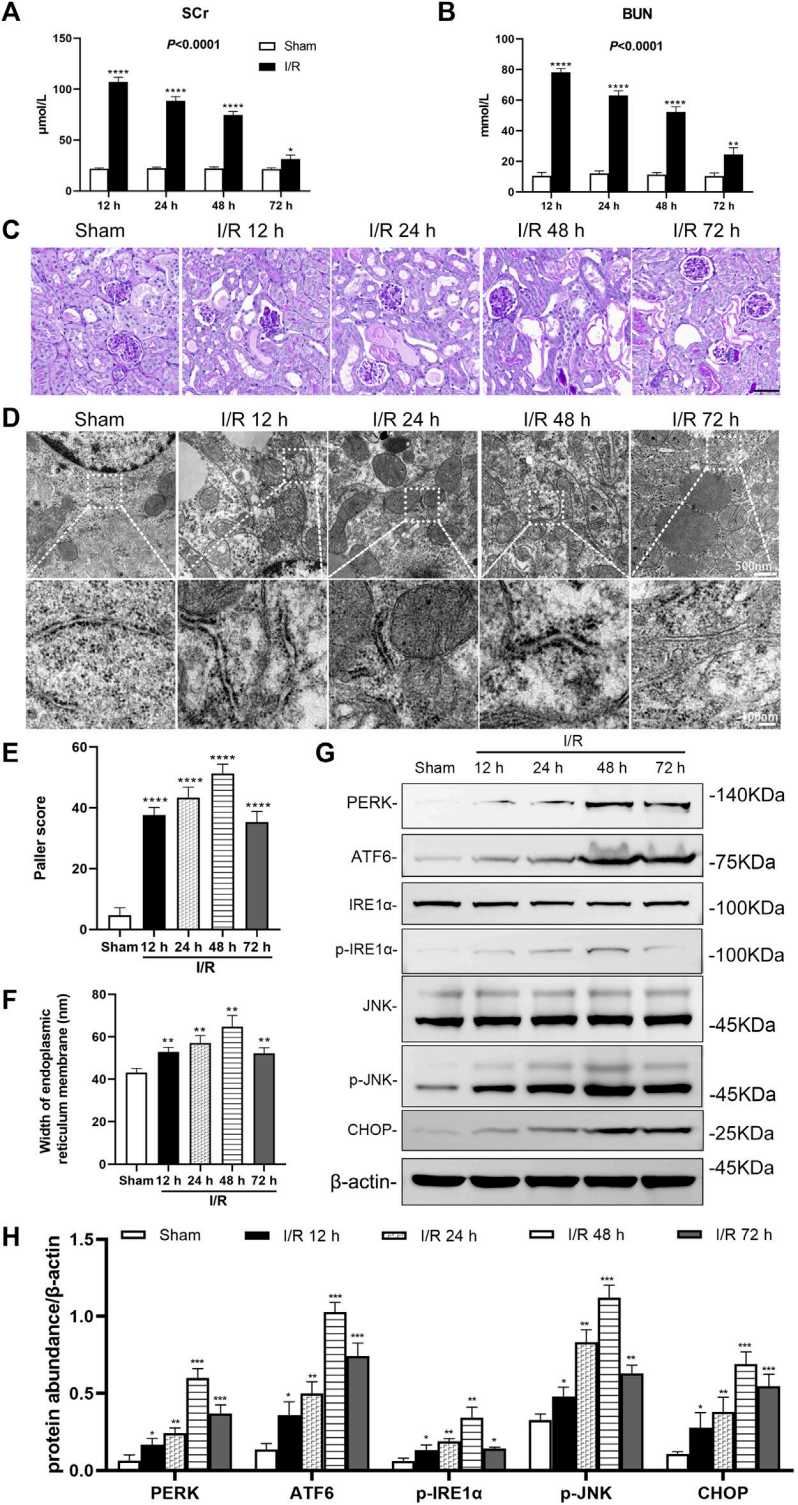
Renal artery ischemia-reperfusion leads to renal insufficiency and ER stress in renal tubules of mice. Male C57BL/6J mice underwent sham operation or bilateral renal artery clamping for 27 min before reperfusion **(A)** Changes in serum creatinine (Scr). When the ischemia-reperfusion (I/R) groups were compared at 12, 24, 48, and 72 h after I/R, the expression was highest at 12 h (*p* < 0.0001) **(B)** Changes in blood urea nitrogen (BUN). When the I/R groups were compared at 12, 24, 48, and 72 h after I/R, the expression was highest at 12 h (*p* < 0.001) **(C)** PAS staining showed lesions in renal tubular tissue. Scale bar = 100 μm **(D)** Transmission electron microscopy was used to observe the ER of mice renal tubules after I/R **(E)** The Paller scores of each group in PAS staining, the score was the highest at 48 h (*p* < 0.0001) **(F)** The analysis of the width of ER in tubular epithelial cells (TEC) of renal tissue, detected by electron microscopy **(G)** Western blotting detection of PERK, ATF6, p-IRE1α, p-JNK, and CHOP in the renal tissue samples, β-actin used as an internal control **(H)** Protein synthesis of ER stress production in western blotting. Due to the death of mice, N = 5 for each group. Statistical significance is denoted as *: *p* < 0.05, **: *p* < 0.01, ***: *p* < 0.001, ****: *p* < 0.0001 compared with the sham group at the same time point.

In the bilateral renal arteries prepared from the I/R group, serum creatinine (Scr), and blood urea nitrogen (BUN) of mice increased over time, peaked at 12 h, then decreased (*p* < 0.0001). Compared with the sham group, the Scr and BUN in the I/R group increased significantly at 12, 24, 48, and 72 h after I/R (at least *p* < 0.05, Figures 1A,B).

PAS staining of renal tissue samples showed that tubule vacuolization and dilation, tubular brush border losses and sloughed, and basement membrane denuded at 12, 24, 48, and 72 h after I/R (Figure 1C). The Paller score showed significant damages to the kidney structure in the I/R groups, compared with the sham group (all: *p* < 0.0001). And the Paller score was more prominent at 48 h (*p* < 0.0001, Figure 1E).

Electron microscopy images showed that in tubular epithelial cells (TEC) an ER endoplasmic granular multivesicular body appeared, and the ER widened after I/R at 12, 24, 48 and 72 h (Figure 1D). Compared with the sham group, the width of ER in the I/R group increased significantly at 12, 24, 48, and 72 h after I/R (all: *p* < 0.01, Figure 1F).

Western blotting analysis showed that I/R induced ER stress in the renal tissue. The large number of ER stress markers (PERK, ATF6, p-IRE1α, p-JNK, and CHOP) increased significantly in the renal tissue at 12, 24, 48, and 72 h after I/R (at least *p* < 0.05, Figure 1G, H).

### I/R renal injury causes ferroptosis in renal tubules of mice

**FIGURE 2:**
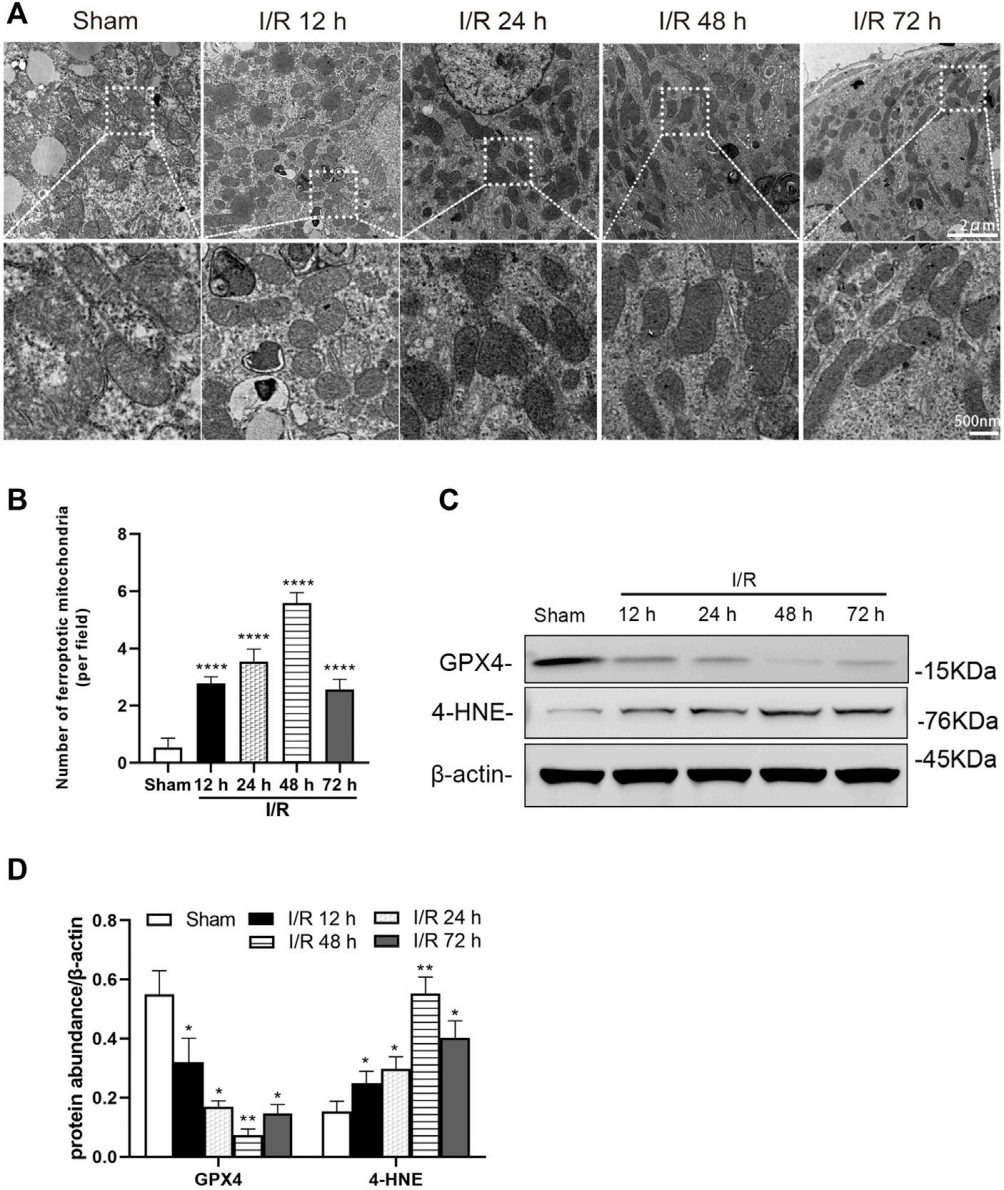
Detection of ferroptosis in the renal tubules of mice at 12, 24, 48, and 72 h after I/R **(A)** Transmission electron microscopy was used to observe the mitochondria of renal tubules **(B)** The numbers of ferroptotic mitochondria per field in different groups were compared **(C)** Western blotting detection of GXP4 and 4-HNE in the renal tissue **(D)** Protein synthesis of GXP4 and 4-HNE in western blotting. Due to the death of mice, N = 5 for each group. Statistical significance is denoted as *: *p* < 0.05, **: *p* < 0.01, ****: *p* < 0.0001 compared with the sham group at the same time point.

Electron microscopy showed evidence of mitochondrion injury in TEC. The images show conditions of shrunken mitochondria, highly condensed mitochondrial membranes and reduced mitochondrial crista at 12, 24, 48, and 72 h after I/R in the I/R group (Figure 2A). The number of ferroptotic mitochondria per field in the I/R group increased significantly at 12, 24, 48, and 72 h after I/R, compared with the sham group (all: *p* < 0.0001). And the number of ferroptotic mitochondria was more prominent at 48 h (*p* < 0.0001, Figure 2B).

Western blotting analysis showed the ferroptosis marker, GPX4 and 4-HNE, were positive in renal tissue, following I/R. At 12, 24, 48, and 72 h after I/R, the protein level of GPX4 was decreased and 4-HNE was increased significantly in the renal tissue of mice from the I/R group (at least *p* < 0.05, Figures 2C,D).

### H/R damage leads to ER stress in HK-2 cells

**FIGURE 3:**
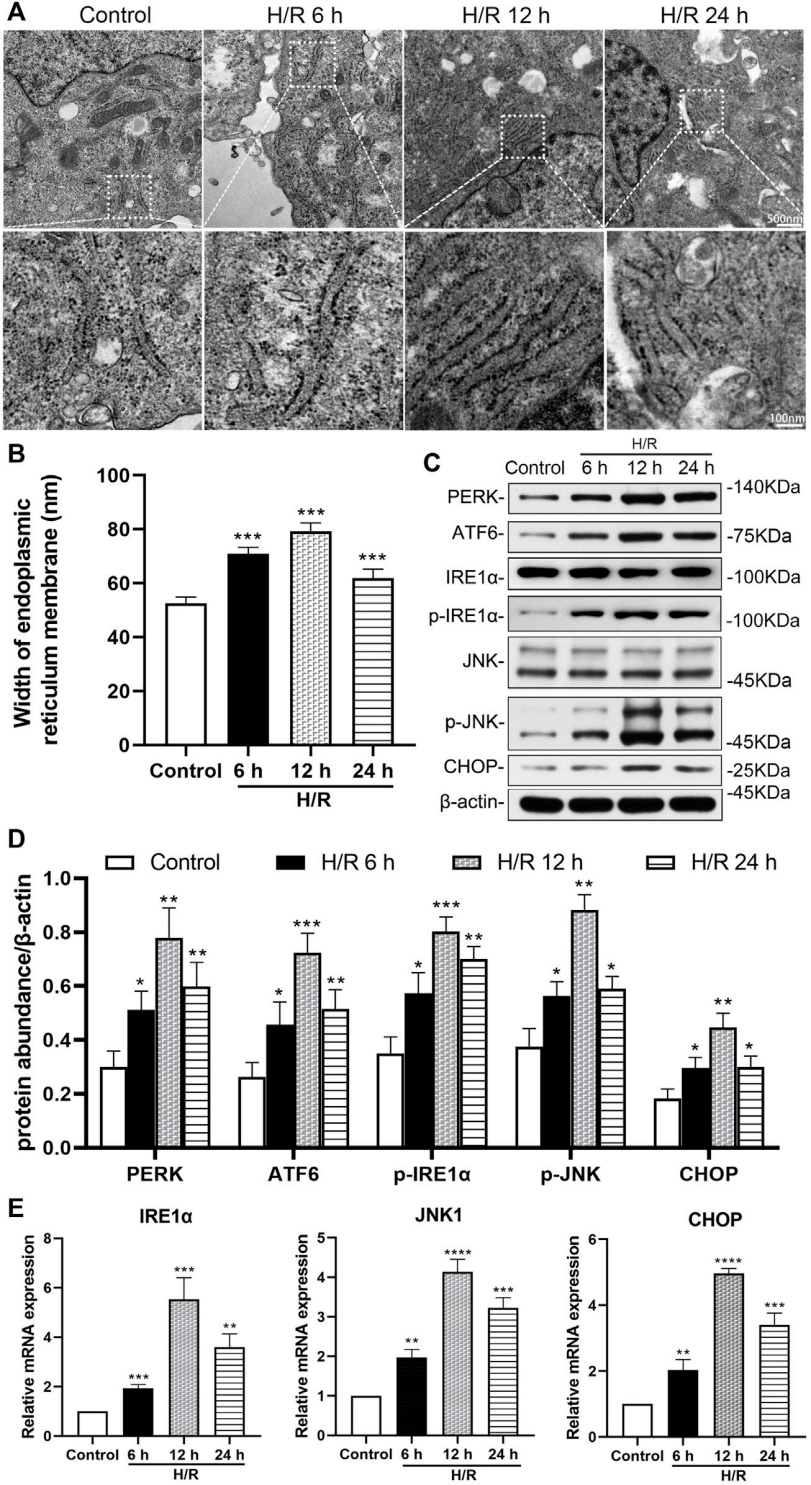
Detection of hypoxia/reoxygenation-induced ER stress in HK-2 cells at 6, 12, and 24 h after hypoxic for 4 h **(A)** Transmission electron microscopy was used to observe the ER of HK-2 cells **(B)** The analysis of the width of ER in HK-2 cells, detected by electron microscopy **(C)** Representative western blotting of ER stress markers **(D)** Protein synthesis of ER stress production in western blotting **(E)** mRNA expressions of IRE1α, JNK1, and CHOP in qRT-PCR. When the hypoxia/reoxygenation (H/R) groups were compared at 6, 12, and 24 h after H/R, the expression was highest at 12 h (*p* < 0.0001). Statistical significance is denoted as *: *p* < 0.05, **: *p* < 0.01, ****: *p* < 0.0001 compared with the control group at the same time point.

The H/R environment triggered an ER stress response in HK-2 cells. Electron microscopy showed that the width of ER in the H/R group increased significantly at 6, 12, and 24 h after H/R,, compared with that in the control group (all: *p* < 0.001, Figures 3A,B).

Western blotting analysis showed the expression of ER stress markers (PERK, ATF6、p-IRE1α, p-JNK, and CHOP) in the H/R group increased significantly at 6, 12, and 24 h of reoxygenation after subjection to hypoxic conditions for 4 h, compared with the control group (at least *p* < 0.05, Figures 3C,D). In addition, qRT-PCR analysis showed that the expression of IRE1α, JNK1 and CHOP mRNA in HK-2 cells increased significantly compared with the control group at 6, 12 and 24 h after H/R (at least *p* < 0.01). And the expressions of IRE1α, JNK1 and CHOP mRNA were more prominent at 12 h (*p* < 0.0001, Figure 3E).

### H/R-induce ferroptosis in HK-2 cells

**FIGURE 4:**
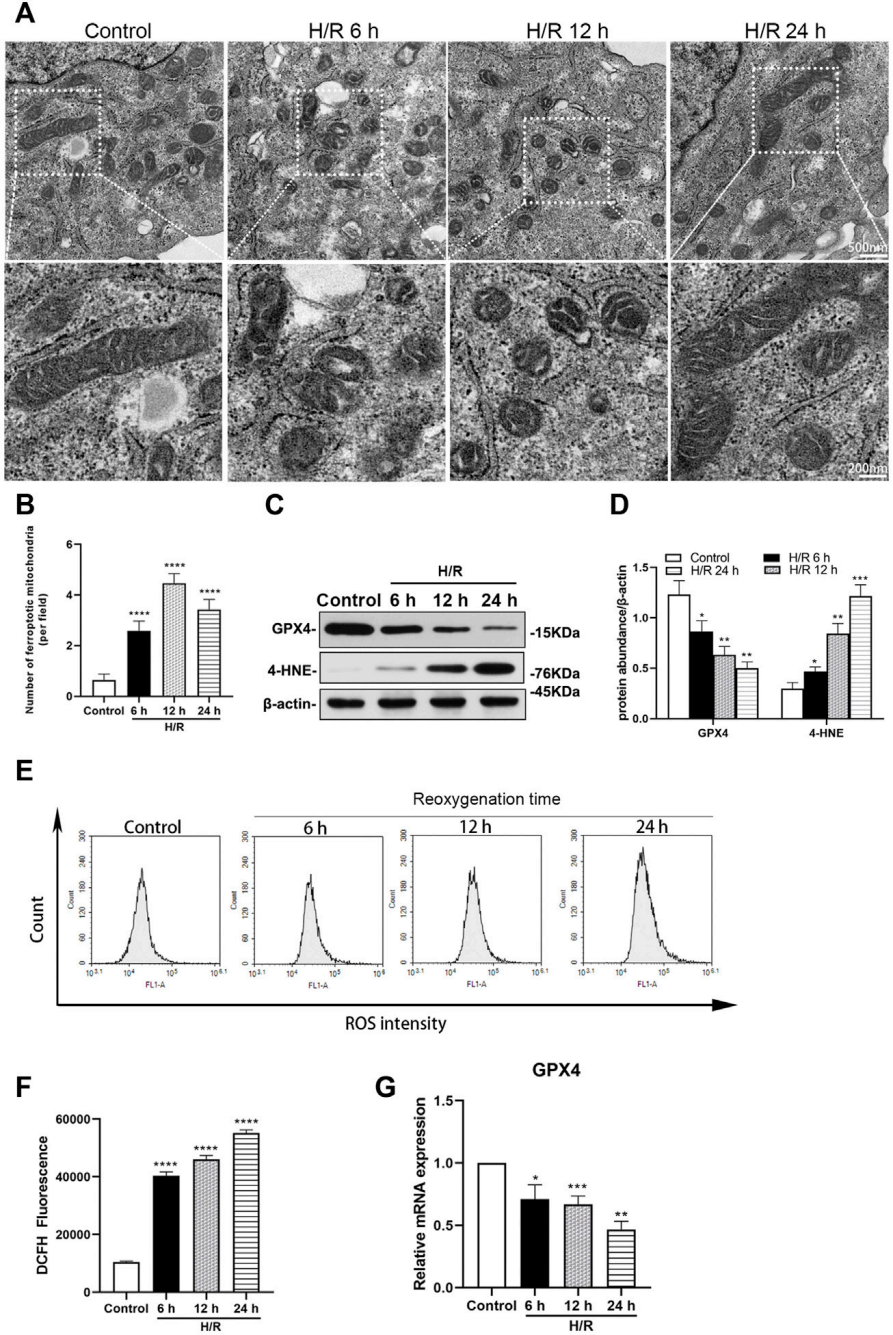
Detection of ferroptosis in H/R HK-2 cells at 6, 12, and 24 h after H/R **(A)** Transmission electron microscopy was used to observe the mitochondrion of HK-2 cells **(B)** The number of ferroptotic mitochondria per field in different groups were compared, the number was highest at 12 h (*p* < 0.0001) **(C)** Representative western blotting of GXP4 and 4-HNE, β-actin used as an internal control **(D)** Protein synthesis of GXP4 and 4-HNE in western blotting **(E)** Flow cytometry showed that the level of reactive oxygen species (ROS) in the control group and H/R HK-2 cells **(F)** Analysis of ROS in flow cytometry in H/R HK-2 cells **(G)** mRNA expressions of GXP4 in qRT-PCR. Statistical significance is denoted as *: *p* < 0.05, **: *p* < 0.01, ***: *p* < 0.001, ****: *p* < 0.0001 compared with the control group at the same time point.

Electron microscopy showed that mitochondria destruction in HK-2 cells was similar with that found in TEC of mice, at 6, 12, and 24 h of reoxygenation following exposure to hypoxic conditions for 4 h (Figure 4A). The number of ferroptotic mitochondria per field in the H/R group increased significantly at 6, 12, and 24 h after H/R, compared with the control group (all: *p* < 0.0001). The number of ferroptotic mitochondria was more prominent at 12 h (*p* < 0.0001, Figure 4B).

Ferroptosis was investigated in HK-2 cells following H/R. The expressions of GXP4 were significantly decreased, while those of 4-HNE were significantly increased in the H/R HK-2 cells, compared with the respective expression levels in the control group (at least *p* < 0.05, Figures 4C,D). Moreover, qRT-PCR analysis revealed a significantly decreased expression of GXP4 mRNA in the H/R HK-2 cells at 6, 12, and 24 h of reoxygenation after exposure to hypoxic conditions for 4 h compared to that of the control group (at least *p* < 0.05, Figure 4G).

The H/R environment increases the ROS produced by HK-2 cells. Flow cytometry analysis showed that, compared with the control group, ROS increased significantly in the H/R group after reoxygenation for 6, 12, and 24 h following exposure to hypoxic conditions for 4 h (all: *p* < 0.0001, Figures 4E,F).

### Inhibition of IRE1/JNK pathway alleviated renal pathological injury in the I/R mice

**FIGURE 5:**
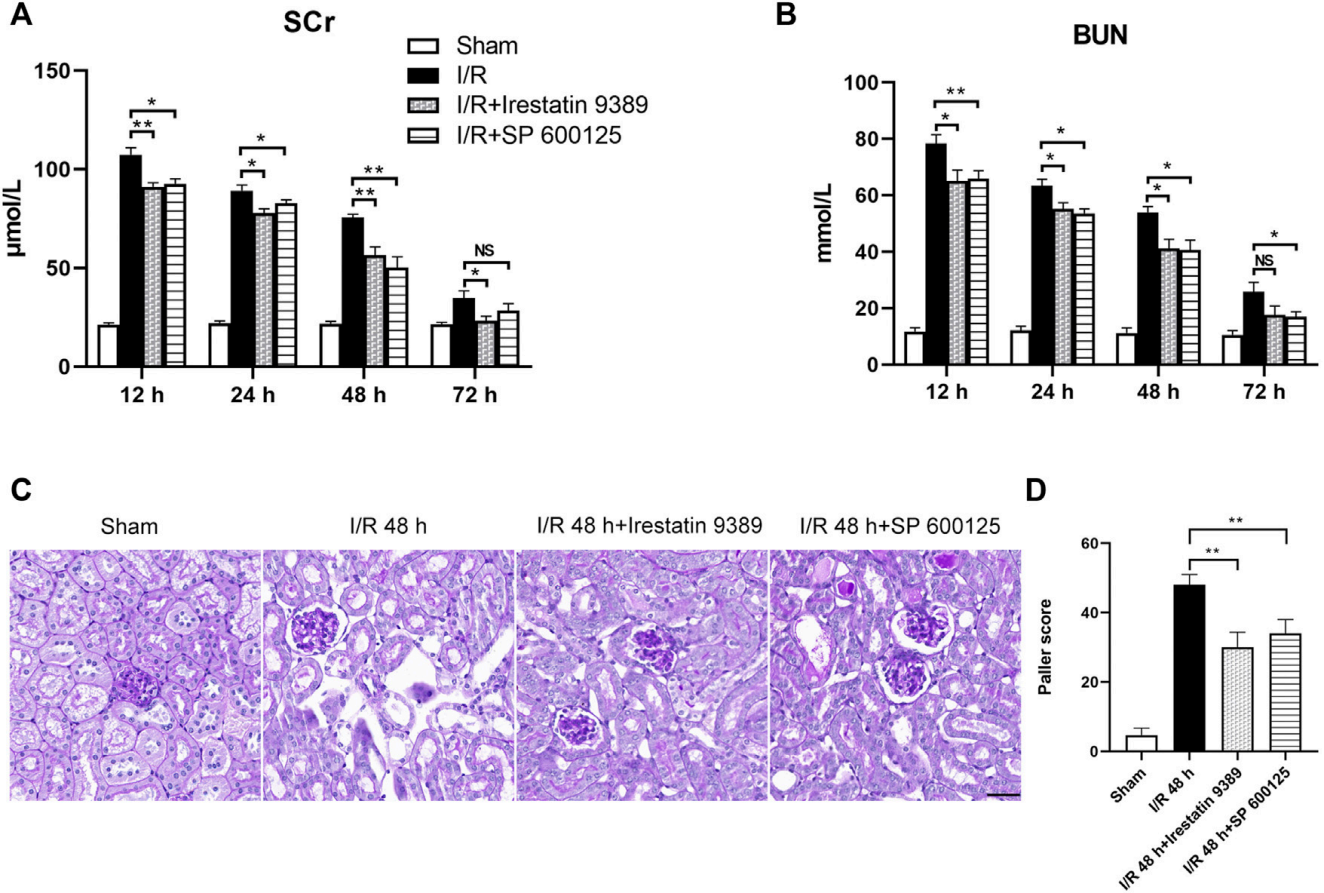
Comparison of renal injury among the sham, I/R, I/R + Irestatin 9389, and I/R + SP 600125 groups. The levels of Scr **(A)** and BUN **(B)** in all four groups at 12, 24, 48 and 72 h after I/R or sham condition **(C)** PAS staining showed the presence of renal tubular lesions in all four groups at 48 h after I/R. Scale bar = 50 μm **(D)** The Paller scores of each group in PAS staining. Results were presented as mean ± SD. Due to the death of mice, N = 5 for each group. Statistical significance is denoted as *: *p* < 0.05, **: *p* < 0.01, NS: *p* > 0.05 in two groups.

Mice were injected with Irestatin 9389 (IRE1 inhibitor) and SP 600125 (JNK inhibitor) respectively, before I/R. The Scr and BUN of both groups decreased significantly at 12, 24 and 48 h after reperfusion, compared with the I/R group (at least *p* < 0.05, Figures 5A,B). PAS staining of renal tissue showed that the injury of TEC in the I/R + Irestatin 9389 and I/R + SP 600125 groups had improved obviously than in the I/R group at 48 h after I/R (all: *p* < 0.01, Figures 5C,D).

### IRE1/JNK pathway regulates ER stress in mice following I/R, and in HK-2 cells following H/R

**FIGURE 6:**
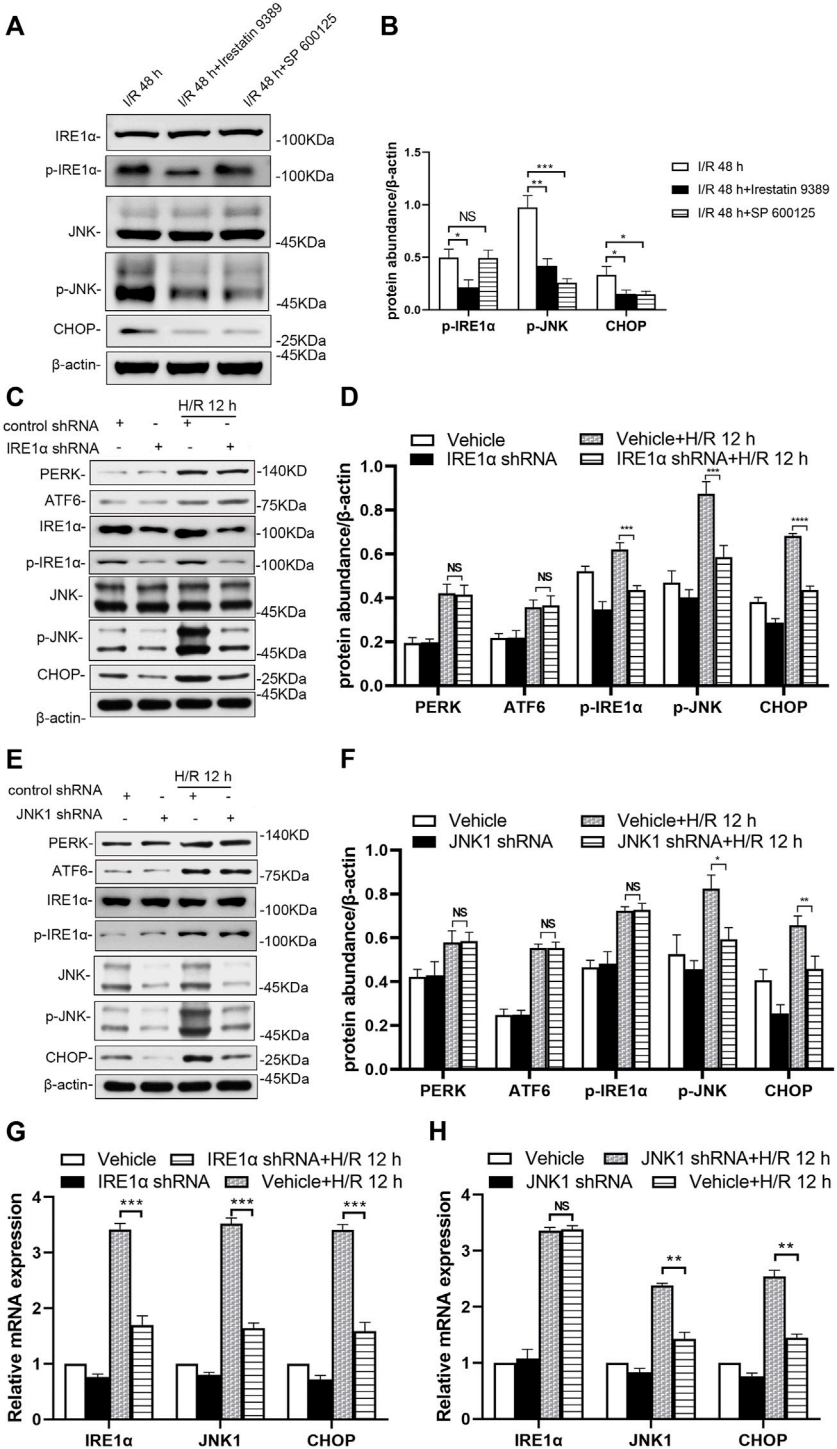
Depletion of IRE1 or JNK reduces ER stress in mice after I/R and HK-2 cells after H/R **(A)** Comparison of p-IRE1α, p-JNK, and CHOP expression in the renal tissue of the I/R, I/R + Irestatin 9389, and I/R + SP 600125 group at 48 h after I/R **(B)** Protein synthesis of p-IRE1α, p-JNK, and CHOP in western blotting in the renal tissue. Due to the death of mice, N = 5 for each group **(C)** Representative western blotting of ER stress markers in the HK-2 cells depleted of IRE1α and infected of vehicle at 12 h after H/R, with β-actin used as an internal control **(D)** Protein synthesis of ER markers in western blotting of the HK-2 cells depleted of IRE1α **(E)** Representative western blotting of ER stress markers in the HK-2 cells depleted of JNK1 and infected of vehicle at 12 h after H/R **(F)** Protein synthesis of ER markers in western blotting of the HK-2 cells depleted of JNK1 **(G)** mRNA expressions of IRE1α, JNK1, and CHOP in the HK-2 cells depleted of IRE1α using qRT-PCR **(H)** mRNA expressions of IRE1α, JNK1, and CHOP in the HK-2 cells depleted of JNK1 using qRT-PCR. Statistical significance is denoted as *: *p* < 0.05, **: *p* < 0.01, ***: *p* < 0.001, ****: *p* < 0.0001, NS: *p* > 0.05 in two groups.

In the group injected with the Irestatin 9389 before I/R in mice, the protein levels of p-IRE1α, p-JNK and CHOP were alleviated significantly in the renal tissue compared with the I/R group at 48 h after I/R (at least *p* < 0.05). In the group injected with the SP 600125 before I/R in mice, the protein levels of p-JNK and CHOP were alleviated significantly in the renal tissue compared with the I/R group at 48 h after I/R (JNK1: *p* < 0.001, CHOP: *p* < 0.05, Figures 6A,B).

IRE1α and JNK1 were knocked-down (shRNA-mediated) to prove their role in the response of HK-2 cells to H/R, especially in ER stress. Western blotting showed knocking down IRE1α significantly down-regulated the expression of IRE1α, as well as that of downstream molecules (p-JNK and CHOP) in the ER stress pathway of HK-2 cells at 12 h after H/R, compared with that in vehicle-infected cells (Figures 6C,D, p-IRE1α: *p* < 0.001, p-JNK: *p* < 0.001, CHOP: *p* < 0.0001). Knockdown of JNK1 in HK-2 cells significantly down-regulated the expression of p-JNK, as well as that of downstream (CHOP) in the ER stress pathway of HK-2 cells at 12 h after H/R (Figures 6E,F, p-JNK: *p* < 0.05, CHOP: *p* < 0.01).

The qRT-PCR results showed that at 12 h after H/R, lentiviral transduction with a shRNA targeting IRE1α significantly down-regulated IRE1α, JNK1 and CHOP in HK-2 cells, compared with that in vehicle-infected cells (Figure 6G, all: *p* < 0.001). A significant decrease in JNK1 and CHOP mRNA levels in JNK1 shRNA-treated HK-2 cells was revealed at 12 h after H/R, compared with that in vehicle-infected cells (Figure 6H, JNK1 and CHOP: *p* < 0.01).

### The IRE1/JNK pathway regulates ferroptosis in mice following I/R and in HK-2 cells following H/R

**FIGURE 7:**
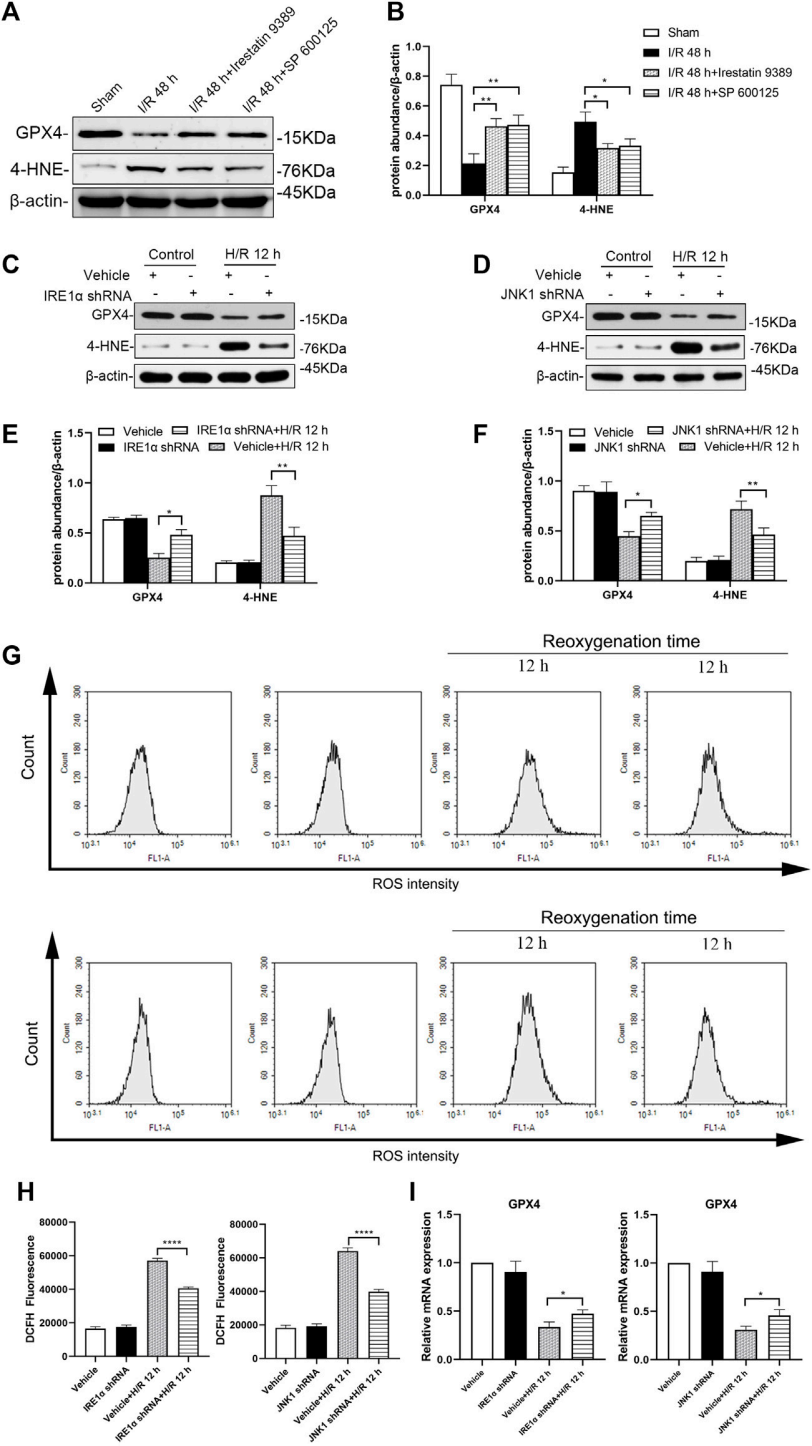
Depletion of IRE1 or JNK reduces ferroptosis in mice after I/R and HK-2 cells after H/R. Western blotting detection **(A)** and protein synthesis **(B)** of GXP4 and 4-HNE in renal tissues of mice at 48 h after I/R. Due to the death of mice, N = 5 for each group. Western blotting was used to detect the expression of GXP4 and 4-HNE in HK-2 cells depleted of IRE1α **(C)** or JNK **(D)** at 12 h after H/R, β-actin used as an internal control. Protein synthesis of GXP4 and 4-HNE in western blotting in HK-2 cells depleted of IRE1α **(E)** or JNK **(F)** at 12 h after H/R **(G)** Flow cytometry detection of ROS produced by HK-2 cells treated with shRNA IRE1α or shRNA JNK1 **(H)** Analysis of ROS in flow cytometry in HK-2 cells at 12 h after H/R **(I)** mRNA expression of GXP4 in HK-2 cells treated with shRNA IRE1α or shRNA JNK1 detected by qRT-PCR. Statistical significance is denoted as *: *p* < 0.05, **: *p* < 0.01, ****: *p* < 0.0001 in two groups.

Western blotting analysis showed that, in mice injected with the Irestatin 9389 before I/R, the protein level of GPX4 was increased and 4-HNE was alleviated significantly in the renal tissue compared with the I/R group at 48 h after I/R (all: *p* < 0.05). Similarly, mice injected with the SP 600125 before I/R, showed increased expression of GPX4 and decreased the protein level of 4-HNE significantly following 48 h after I/R (GXP4: *p* < 0.05, 4-HNE: *p* < 0.01, Figures 7A,B).

Western blotting analysis showed that the production of GPX4 in HK-2 cells treated with IRE1α shRNA increased while that of 4-HNE decreased significantly at 12 h after H/R, compared with vehicle-infected HK-2 cells (Figures 7C,E, GXP4: *p* < 0.05, 4-HNE: *p* < 0.01). Similarly, compared with vehicle-infected HK-2 cells, JNK1 shRNA treated HK-2 cells showed the increased GPX4 and decreased 4-HNE expressions at 12 h after H/R (GXP4: *p* < 0.05, 4-HNE: *p* < 0.01, Figures 7D,F).

The production of ROS in HK-2 cells treated with IRE1α shRNA, following exposure to hypoxic conditions for 4 h and subsequent reoxygenation for 12 h, was significantly reduced, compared with that of the vehicle-infected HK-2 cells (*p* < 0.0001). Similarly, the production of ROS in HK-2 cells treated with JNK1 shRNA was also significantly reduced, compared with that of the vehicle-infected HK-2 cells, at 12 h after H/R (*p* < 0.0001, Figures 7G,H).

The qRT-PCR results showed that, compared with the vehicle-infected cells, the GPX4 mRNA expression level in HK-2 cells treated with IRE1α shRNA or JNK1 shRNA was significantly higher (Figure 7I, all: *p* < 0.05).

### Inhibition of ferroptosis attenuates ER stress including the IRE1/JNK pathway

**FIGURE 8:**
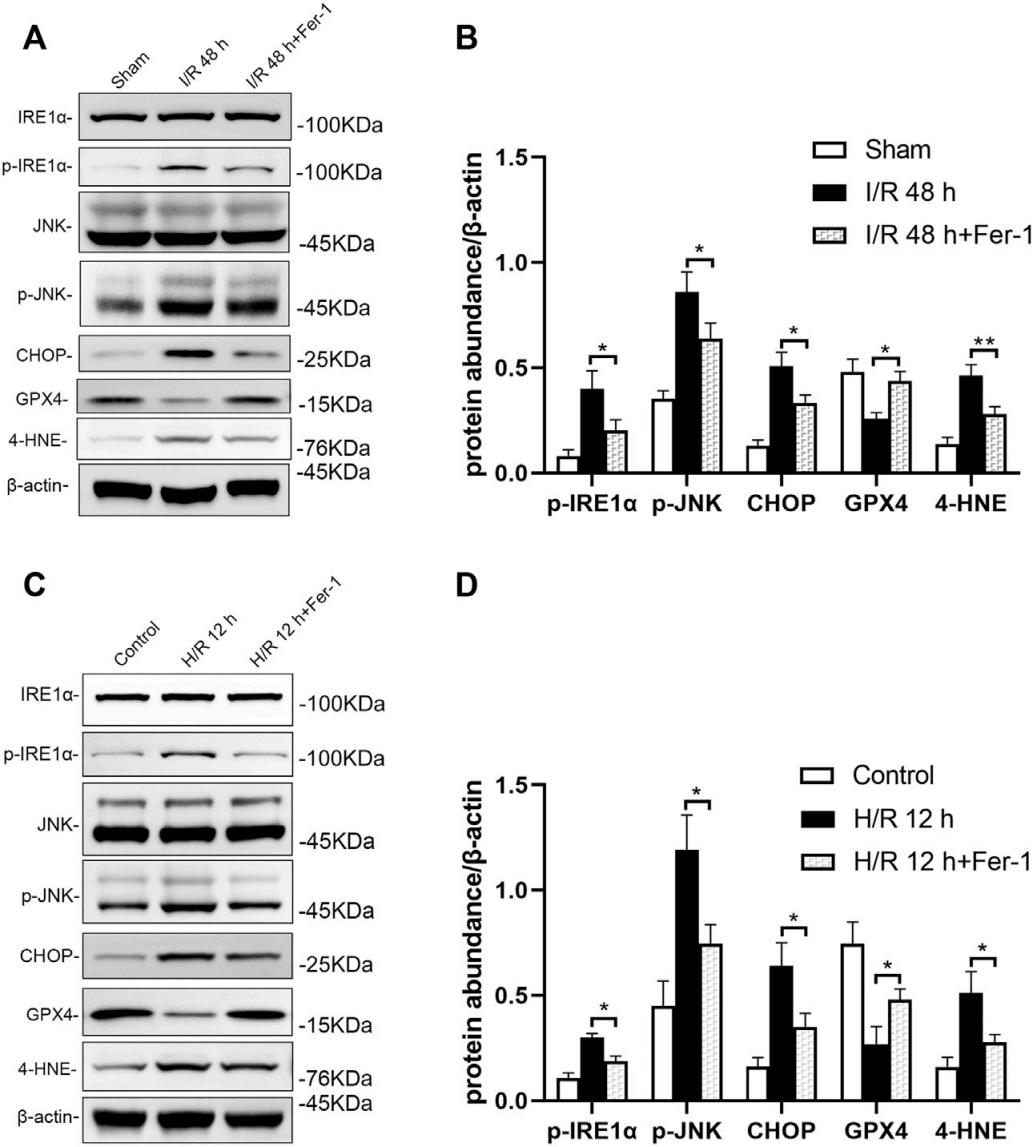
Inhibition of ferroptosis attenuates IRE1/JNK pathway in mice after I/R and HK-2 cells after H/R. Western blotting detection **(A)** and protein synthesis **(B)** of p-IRE1α, p-JNK, and CHOP in mice at 48 h after I/R. Due to the death of mice, N = 5 for each group. Western blotting detection **(C)** and protein synthesis **(D)** of p-IRE1α, p-JNK, and CHOP in HK-2 cells at 12 h after H/R. Statistical significance is denoted as *: *p* < 0.05, **: *p* < 0.01 in two groups.

Inhibition of ferroptosis could reduce ER stress including the IRE1/JNK pathway in renal tissue of mice following I/R. Western blotting showed that p-IRE1α, p-JNK, CHOP, and 4-HNE levels were decreased significantly, while GPX4 level was elevated significantly in I/R + Fer-1 group, compared with the I/R group at 48 h after I/R (at least *p* < 0.05, Figures 8A,B). Similar, intervened by Fer-1, p-IRE1α, p-JNK, CHOP, and 4-HNE levels in HK-2 cells were decreased significantly, GXP4 level was increased significantly, compared with those in the H/R group at 12 h after H/R (all: *p* < 0.05, Figures 8C,D).

## Discussion

Despite medical progress and prevention efforts in recent years, the high incidence of AKI has made it a global priority in public health. In China, the detection rate of AKI was 2.03% by expanded criteria (Yang et al., 2015). It is estimated that about 1.4 million patients with AKI are estimated to have been treated in hospital in China during 2013, consuming about US$13 billion for their entire in-hospital cost around 10% of China’s total medical expense (Yang et al., 2015). AKI is characterized by the injury and death of renal tubular cells, making tubular repair and regeneration the primary processes to induce kidney recovery from AKI (Bonventre and Yang, 2011; Sharfuddin and Molitoris, 2011; Zarjou and Agarwal, 2011; Kinsey et al., 2013). Characteristic morphological and pathological changes in cells affected by AKI include organelle lesions, ER stress, mitochondrial ROS accumulation, inflammatory response, apoptosis, ferroptosis, and other forms of cell death (Muller et al., 2017). In our study, we found that the Scr and BUN of mice increased significantly, in the renal artery of I/R AKI model of mice, compared with the sham group. For the pathological morphology, the primary type of lesion in renal tissue were injuries in the renal tubule. The TEC in the I/R mice model were obviously shed, the basement membrane was exposed, the Paller score of renal tubules was significant damages, and the ROS in HK-2 cells increased significantly after H/R, compared with the control group. Electron microscopy further showed that endoplasmic granular multivesicular bodies appeared, the width of ER increased significantly, mitochondria shrunk, condensed mitochondrial membranes, and the number of ferroptotic mitochondria per field increased significantly in renal tubules of renal I/R mice and H/R HK-2 cells (Figures 1–4). These results suggest that TEC were damaged in I/R induced AKI, and the ER and mitochondria in these cells were injured.

Ferroptosis is a type of regulated, programmed cell death recently identified as an iron and lipid hydroperoxide-dependent nonapoptotic cell death. Ferroptosis is present in some models of renal injury, such as I/R induced and folic acid induced AKI (Martin-Sanchez et al., 2017). Ferroptosis can be induced by the down-regulation of system Xc− activity, inhibition of GPX4, and an increase in production of lipid ROS (Liu et al., 2020). GPX4, a glutathione-dependent enzyme, is a key regulator of ferroptosis and subsequent intracellular accumulation of lipid ROS (Yagoda et al., 2007; Dixon et al., 2012; Yang et al., 2014; Zhang et al., 2021). Mitochondria are the major source of cellular ROS; however, ROS can damage mitochondrial membrane lipids, leading to impaired mitochondrial functions (Paradies et al., 2002). Accumulation of ROS then activates the process of ferroptosis (Shimada et al., 2016). 4-HNE is a production of oxidation of lipids such as cardiolipin in ferroptosis (Yoo et al., 2012; Yamada et al., 2020). Reduced level of GPX4 and increased level of 4-HNE are always regarded as markers of ferroptosis. In our study, we found that expression levels of GPX4 were clearly decreased and those of 4-HNE were increased significantly in the TEC after AKI in I/R induced mice and H/R induced HK-2 cells. Binding ROS increased significantly in H/R HK-2 cells. Therefore, ferroptosis in AKI is likely induced by I/R or H/R.

ER stress has long been considered a promoting factor in AKI. The stress triggers the unfolded protein response in the ER, which involves three ER stress sensors, and double stranded RNA activated PERK, ATF6, and IRE1 (Zuk and Bonventre, 2016). The activation of IRE1 stimulates the JNK pathway, which can inflict further damage (Bonventre and Zuk, 2004). In our study, we found wide spread expression of ER stress markers ATF6, PERK, p-IRE1、p-JNK and CHOP that were markedly increased in the renal tissue of I/R mice and TEC following H/R. Additionally, IRE1 regulated downstream JNK and CHOP, whose expression levels increased significantly. We also found that the IRE1/JNK pathway could regulate renal function damage in mice as well as pathological damages including ER stress *in vivo* and *in vitro* experiments.

Recent evidence has shown that ER stress signaling has close cross-talk with ferroptosis. Specifically, ER stress disrupts Ca^2+^ homeostasis in the ER, further causing mitochondrial calcium overload and increasing ROS production (Shimada et al., 2016). The unfolded protein response is then caused by ferroptosis inducers (De Stefani et al., 2012). Meanwhile, ferroptosis could promote the cystine–glutamate antiporter system Xc-, which may induce further ER stress (Rahmani et al., 2007; Sauzay et al., 2018). ER stress can also be triggered by ROS, which are also primary promoters of ferroptosis (Sun et al., 2015). Additionally, ferroptosis agonists sorafenib, erastin, and sulfasalazine, could activate the ATF4-CHOP pathway (Cao and Kaufman, 2014). Erastin specifically might specifically stimulate JNK, and the ATF4-CHOP pathway could be inhibited by JNK inhibitor SP 600125 (Xie et al., 2016). In our study, SP 600125 treated I/R mice were protected from functional AKI, evidenced by the content of JNK in renal tissue decreasing significantly, morphological improvement of mitochondria, 4-HNE expression decreasing, and GXP4 increasing significant, compared with the I/R group. In JNK1 knock-down HK-2 cell lines, following the changes above, ROS accumulation reduced significantly. Meanwhile, the inhibitor of IRE1 treated I/R mice and H/R HK-2 cells also demonstrated the protective effect of inhibiting ferroptosis (Figures 6, 7). Especially, we observed that inhibition of IRE1/JNK pathway induced the renal function and the renal tubular injuries of the I/R mice reduced significantly (Figure 5). These data suggest that inhibition of the IRE1/JNK pathway could protect against I/R and H/R induced TEC injuries, partly by inhibiting ferroptosis in AKI. In addition, in order to know whether ferroptosis has effect on ER stress, we used Fer-1, an inhibitor of ferroptosis, to intervene in mice before I/R and HK-2 cells before H/R. The results showed that inhibition of ferroptosis could also attenuate ER stress, including the IRE1/JNK pathway. Therefore, there is a cross-talk between IRE1/JNK pathway and ferroptosis (Figure 8).

This study has scope for improvements. The pathophysiological changes of AKI are very complex. Some studies have found a close relationship between apoptosis and ferroptosis (Garg and Vucic, 2016; Xie et al., 2016; Lee et al., 2018). This study detected only some of mechanisms, possibly of many, which still needs to deeper study. In our previous study, the IRE1/JNK pathway mediated production of IL-6 and MCP-1 in HK-2 cells following H/R (Liang et al., 2020). Meanwhile, some reports showed a link between ferroptosis and inflammation (Sun et al., 2020). Probably, the IRE1/JNK pathway affects ferroptosis by inflammation. And that, we further suggest that ER stress is inextricably linked with apoptosis, ferroptosis and a variety of pathways, which should be further investigated. Overall, intervention in future preclinical studies in animal disease models, followed by well-designed clinical trials, should show the true therapeutic potential of targeting these pathways. However, the relationship between the IRE1/JNK pathway and ferroptosis in AKI requires validation of these conclusions and further study.

## Conclusion

Several *in vitro* and *in vivo* studies have revealed that ferroptosis plays an important role in I/R and H/R induced AKI, which causes damage of renal tubules. The inhibition of the IRE1/JNK pathway can protect against I/R and H/R induced TEC injury by inhibiting ferroptosis. There is also a cross-talk between the IRE1/JNK pathway and ferroptosis. These findings suggest potential biomarkers as therapeutic targets for the prevention and treatment of AKI.

## Data Availability

The datasets presented in this study can be found in online repositories. The name of the repository can be found below: https://www.jianguoyun.com/p/Daj4-bgQutDIChi-wNAEIAA.

## Abbreviations

AKI, acute kidney injury; IRE1, Inositol requiring enzyme 1; JNK, c-Jun NH2-terminal kinases; ER, endoplasmic reticulum; I/R, ischemia-reperfusion; ROS, reactive oxygen species; H/R, hypoxia/reoxygenation; Fer-1, ferrostatin-1; PBS, phosphate buffer saline; PAS, Periodic Acid-Schiff; PERK, protein kinase -like endoplasmic reticulum kinase; ATF6, activating transcription factor 6; CHOP, CCAAT-enhancer-binding protein-homologous protein; 4-HNE, 4-hydroxynonenal; GPX4, glutathione peroxidase 4; FBS, fetal bovine serum; DMEM, Dulbecco’s Modified Eagle Medium; qRT-PCR, quantitative reverse transcription-polymerase chain reaction; DCFH-DA, 2,7-Dichlorodi-hydrofluorescein diacetate; Scr, serum creatinine; BUN, blood urea nitrogen; TEC, tubular epithelial cells.
